# Prioritizing Positivity, Styles of Rumination, Coping Strategies, and Posttraumatic Growth: Examining Their Patterns and Correlations in a Prospective Study

**DOI:** 10.3389/fpsyg.2022.842979

**Published:** 2022-02-25

**Authors:** Mariusz Zięba, Katarzyna Wiecheć, Natalia E. Wójcik, Michał J. Zięba

**Affiliations:** ^1^Center for Trauma, Crisis and Growth, SWPS University of Social Sciences and Humanities, Poznań, Poland; ^2^Department of Clinical Psychology, Poznań University of Medical Sciences, Poznań, Poland

**Keywords:** prioritizing positivity, posttraumatic growth, life satisfaction, hope, hierarchical regression analysis

## Abstract

**Objectives:**

A growing number of studies indicate that coping with the experience of a crisis event, which causes a breach in the individual’s fundamental beliefs regarding the world and his/her place in it, can result in posttraumatic growth. Positive emotions can have an undoing effect on negative emotional arousal and broaden an individual’s scope of action, and they can foster posttraumatic growth. This study aimed to examine relations between prioritizing positivity, styles of rumination, coping strategies, and posttraumatic growth.

**Methods:**

One hundred and sixty-four Polish adults took part in the study, filling out questionnaires to measure prioritizing positivity, hope, and self-efficacy. Twelve to fifteen months later, 104 of them accepted the invitation to the second part of the study. The participants reported the intensity of rumination associated with the most critical event in their lives, which took place between the first and second stages of the study and the coping strategies they used. Posttraumatic growth and life satisfaction were also measured.

**Results:**

Results from hierarchical regressions found that higher levels of prioritizing positivity, deliberate ruminations, and religious coping and lower level of intrusive ruminations were associated with posttraumatic growth. The results also indicate that self-esteem was a significant predictor of life satisfaction.

**Conclusions:**

The results of the prospective study confirm that individual differences in prioritizing positivity can relate to a process of posttraumatic growth. Prioritizing positivity was associated with the use of an active coping strategy and deliberate but not intrusive ruminations. Previous studies on the role of prioritizing positivity have focused on the impact on the level of wellbeing of seeking positive emotions in everyday life. Our results show the importance of prioritizing positivity in coping with stress and trauma. These results can be used to design effective psychological intervention techniques to support people experiencing trauma and psychological crises. The results also indicate that life satisfaction has different predictors from posttraumatic growth.

## Introduction

A growing number of studies indicate that the process of coping with the experience of a traumatic or crisis event, which causes a breach in the individual’s assumptive world and fundamental beliefs (Janoff-Bulman, 1992, 2004), can result in posttraumatic growth (Linley and Joseph, 2004; Tedeschi et al., 2018; Taku et al., 2021). Posttraumatic growth has been defined as positive psychological changes experienced as a result of the struggle with the aftermath of a highly stressful and potentially traumatic life event that may be observed in five domains: increasing the sense of personal strength, improving relationships with other people, discovering new opportunities, heightening appreciation for life, and spiritual and existential changes (Taku et al., 2008; Shakespeare-Finch et al., 2013; Tedeschi et al., 2017, 2018). The theoretical model of posttraumatic growth (Tedeschi et al., 2018) includes pre-trauma factors (demographic characteristics, individual differences, mental status and pre-trauma assumptive world, and core beliefs), characteristics of the potentially disruptive (seismic) event, challenges to core beliefs, ruminative thoughts, managing emotional stress and coping, and self-disclosure. All these elements are interconnected and can interact.

The authors of the concept of posttraumatic growth refer to the definition of trauma presented by Janoff-Bulman (1992) as an event that represents significant challenges to individuals’ ways of understanding the world and their place within it (Tedeschi and Calhoun, 2004). Such an event undermines person’s previously positive core beliefs that the world is benevolent and meaningful and that the self is worthy. Therefore, the direct result of a seismic event is at least a temporary questioning of beliefs with which the experience of trauma is inconsistent. Faced with such an experience, a person puts effort into cognitive processing. Joseph and Linley (2005) and Payne et al. (2007) propose that the Piagetian terms “assimilation” and “accommodation” could be used to describe the after-trauma cognitive processes. Assimilation occurs when an event is interpreted as substantially consistent with a person’s existing cognitive schemas, so no significant change in these schemas is necessary. Among the assimilation processes, Joseph and Linley (2005) also include attempts to ignore the event and avoid thinking about it. Accommodation means the process of changing one’s core beliefs under the influence of an event. These changes can be positive or negative, leading to posttraumatic growth or posttraumatic depreciation (Baker et al., 2008; Cann et al., 2010; Taku et al., 2021).

Tedeschi and Calhoun (2004) and Tedeschi et al. (2018) use the term “ruminations” to describe the cognitive efforts to deal with the discrepancy in assessing a traumatic event and fundamental beliefs, distinguishing between two types: intrusive and deliberate ruminations (Cann et al., 2011), and assigning to each of them a different role in the process of posttraumatic growth. In the initial stage of posttraumatic re-adaptation, ruminations are automatic and intrusive, affecting the persistence of tension and stress (Cann et al., 2010, 2011). For many people, these ruminations gradually become more reflective (deliberate). A cognitive engagement replaces intrusive thoughts with reflective thinking about the traumatic experience and its consequences—what happened and what it can mean. Deliberate (reflective) ruminations result from efforts to understand and reinterpret the trauma, and they are to a greater extent conscious and often intentional. The intensity and persistence of intrusive rumination are predictors of posttraumatic depreciation and deliberate rumination of posttraumatic growth (Tedeschi et al., 2018; Ogińska-Bulik and Michalska, 2021; Taku et al., 2021).

Neither posttraumatic growth nor depreciation result from experiencing trauma or a crisis event. The development of posttraumatic growth could result from emotional and cognitive coping with a traumatic experience and its impact on the functioning of a person. An essential element of the posttraumatic growth process is managing emotional distress, which may be fostered by cognitive involvement in the processing of the experience, the use of adequate coping strategies (Bussell and Naus, 2010; Rajandram et al., 2011), and above all, disclosure and use of social support (Calhoun and Tedeschi, 2013; Nordstrand et al., 2020). The positive changes perceived by the trauma survivor seem to be related to assessing the person’s actions in the face of the trauma. A sense of increased personal strength is more likely to occur when someone judges that they have coped, perhaps better than they might previously have guessed, with a trauma or crisis event and its consequences. Positive changes in terms of discovering new opportunities, amplifying appreciation for life, and improving relationships with other people, seem possible when a person makes appropriate changes in their daily functioning. Spiritual and existential changes may concern people who use religious coping. Different coping strategies may support posttraumatic growth differently in its different areas. However, it should also be remembered that even the most effective coping with stress does not lead directly to the development of posttraumatic growth.

Many studies show that inducing positive emotions when confronted with severe life challenges and crises supports coping strategies (Folkman and Moskowitz, 2000a,b; Fredrickson, 2004; Tugade et al., 2004; Folkman, 2008; Lelorain et al., 2010, 2012). According to the Fredrickson’s broaden-and-build theory (Fredrickson 1998, 2001; Fredrickson and Branigan, 2005), positive emotions effectively reduce tension and stress, broaden the scope of one’s attention and thinking, and build personal resources, such as adequate coping strategies. Moreover, the results of a longitudinal study conducted by Fredrickson et al. (2003) suggest that a high level of positive emotions before the traumatic event predicts posttraumatic growth. According to Norlander et al. (2005), too, a high level of positive emotions in daily life is associated with posttraumatic growth. Personality traits, such as high levels of extraversion and low neuroticism, are a factor in how often people experience positive emotions in their daily lives (Costa and McCrae, 1980; Steel et al., 2008). Moreover, prioritizing positivity, defined as an individual difference that reflects the ability to seek pleasant states in everyday activities, can also relate to experiencing positive emotions (Catalino et al., 2014; Catalino and Boulton, 2020; Machlah and Zięba, 2021).

The role of fundamental beliefs about oneself, other people and the world in the process of posttraumatic growth is complex. On the one hand, these beliefs challenged by the experience of trauma, and then they rebuilt (Janoff-Bulman, 2004; Tedeschi et al., 2018). On the other hand, pre-trauma beliefs can influence one’s ability to manage a traumatic experience. Positive beliefs about the future, such as optimism and hope, are understood in psychology in many different ways. Not all kinds of such positive expectations are conducive to adaptation to a life crisis (Aspinwall and Tedeschi, 2010). Among the positive beliefs that can play a particularly positive role in the process of posttraumatic growth, it is worth mentioning hope (Snyder, 2002) and basic trust (Trzebiński and Zięba, 2004). According to Snyder (2000, 2002) and Snyder et al. (1991), hope is a motivational state based on two interrelated beliefs—agency and pathways. Agency is a goal-directed determination and the perceived ability to reach desired goals. Pathways thinking is the perceived ability to produce plausible routes to the goals. Agency and pathways components enhance one another and are affected by each other (Snyder, 2000). Hope is related to positive affect and more positive thoughts (Snyder, 2002), psychological adjustment (Kwon, 2002), and the use of adaptive coping strategies (Gum and Snyder, 2002). Basic trust is a fundamental assumption that the world has unchangeable order and meaning and is generally positive toward human beings (Trzebiński and Zięba, 2004). The results of previous studies on oncology patients indicate that level of basic trust is positively related to the posttraumatic growth (Trzebiński and Zięba, 2013).

This study aimed to examine relations between prioritizing positivity, styles of rumination, coping strategies, and posttraumatic growth. According to the previous studies, prioritizing positivity is associated with a high overall level of life satisfaction and less depression, better positive relationships with others, ego-resilience, self-compassion, and mindfulness (Catalino et al., 2014; Catalino and Boulton, 2020; Machlah and Zięba, 2021). It is known from the research results presented above that prioritizing positivity is conducive to taking active measures that increase the probability of experiencing positive emotions. Based on current knowledge about the role of positive emotions in posttraumatic growth, we consider that prioritizing positivity can act as a personal resource. When struggling with life crises, people with a high level of prioritizing positivity experience positive emotions more often. This, in turn, may affect the use of more adaptive coping strategies and the cognitive processing of difficult life experiences. Positive emotions can contribute to a higher openness to finding solutions and discovering new ways of acting and interpreting experience. However, no studies have so far been carried out to check the potentially positive role of prioritizing positivity in the context of traumatic or crisis experiences. The study also aimed to check whether and to what extent the potentially positive effect of prioritizing positivity on posttraumatic growth is mediated by the intensification of deliberate ruminations and the use of adaptive coping strategies. The hypothesis that the positive role of prioritizing positivity in the process of posttraumatic readaptation is related to the level of hope and basic trust was also subject to empirical verification. We further checked to what extent the paths leading to posttraumatic growth and experiencing life satisfaction differ.

## Materials and Methods

### Participants

The participants of the first study stage were 164 adult residents of Poznań (a large city in the western part of Poland) and the surrounding area who responded to a request to participate in a research project on life events. The invitation was spread on local websites and leaflets distributed throughout the city. In the period from 12 to 16 months later, we recontacted the participants to recruit them for the next stage of the study. A portion of the first stage sample could not be reached by email because addresses were unavailable or no longer valid (11 of 164, or 6.7%). Of the others, 120 expressed an interest in participating and 104 of them eventually did so, representing a 63.4% response rate. The second stage study participants (*N* = 104) did not differ from those who did not participate (*n* = 60) in age, sex, or any of the variables reported in the first stage of the study. The eventual sample included 66 women and 38 men with ages ranging from 19 to 62 years (*M* = 30.98, *SD* = 9.66). In terms of marital status, 38 were single, 45 in an informal relationship, 16 married, four divorced, and one not reported. Fifty-three participants finished school beyond the high school level, 48 finished high school and three reported an education level of “other”. Ninety-one (87.5%) participants were currently employed. The variety of occupations in the sample was large, and none of the occupational groups included more than a few participants.

### Procedure

The data analyzed in this paper come from a larger research project carried out at the Research Center for Trauma, Crisis and Growth (Poland). This project includes three stages over several years. Its main scientific goal is to verify the hypotheses regarding the impact of the narrative representation of experience on coping with trauma or difficult life events. At each stage of the study, participants take part in a psychological interview. At the first meeting, an interview was conducted in the Life Story Interview (McAdams, 2007). The interviewees related eight important scenes they selected from their lives. At the next meetings, interviews concerned traumatic or crisis events that took place between the first and second stages of the study and their influence on the fundamental beliefs of the interviewee. In the present article, we do not analyze the data collected in the interviews but focus on quantitative data.

The presented data come from two stages of the study. In the first, the participants filled out questionnaires measuring prioritizing positivity, basic trust, hope, and self-esteem. About half of the questionnaire sets were completed in the laboratory in a paper-and-pencil version. Due to the limitations resulting from the COVID-19 pandemic, data collection was then continued online on the Qualtrics platform.

The second stage of the study took place after 12 to 15 months. Firstly, the participants talked about their most difficult life events during that period. The events reported were 17.3% relationship problems (e.g., betrayal or breakup; *n* = 18), 14.4% serious medical event or injury (*n* = 15), 10.6% serious medical event or injury for close other (*n* = 11), 10.6% COVID-19 pandemic (lockdown, quarantine; *n* = 11), 10.6% family problems (*n* = 11), 9.6% problems at work (*n* = 10), 7.7% job loss (*n* = 8), 5.8% unexpected death of close other (*n* = 6), and 13.5% various others (*n* = 14).

Within days of the interview, study participants completed questionnaires to measure their coping strategies and ruminations related to the previously reported event. Then they filled out scales to measure various aspects of the impact of that event and coping with it on their current functioning: anxiety and depression experienced in the last weeks, life satisfaction, and posttraumatic growth. The measurement was conducted online on the Qualtrics platform.

The participants had received information about the procedure and the Informed Consent Form before the interview, and they could withdraw from the study at any time. The university ethical committee approved the study. For participation in each stage of the study, participants received remuneration in the form of a shopping voucher worth PLN 50 (about EUR 12).

### Measures

#### Measures in the First Stage of the Study

##### Prioritizing Positivity

We used the Polish version (Machlah and Zięba, 2021) of the Prioritizing Positivity Scale (Catalino and Boulton, 2020). This scale includes five statements that measure whether people organize their time to maximize their positive emotions. The Cronbach’s *α* of the questionnaire in the study was 0.77.

##### Basic Trust

Basic trust was measured using an eight-item scale (Trzebiński and Zięba, 2004). Participants indicated their belief in two world characteristics: its higher-order and sense, and its general positivity toward human beings. Participants provided their ratings using a five-point scale (1 = strongly disagree, 5 = strongly agree). Cronbach’s *α* for the scale was 0.81.

##### Hope

We used the Polish version (Łaguna et al., 2005) of the Adult Dispositional Hope Scale (Snyder et al., 1991), which measures hope in terms of how people perceive themselves when pursuing a goal in different situational contexts. This questionnaire contains eight statements—four measure agencies and the other four measure pathways thinking. Each of the items was rated on an eight-point Likert scale ranging from “1 = definitely false” to “8 = definitely true.” Cronbach’s *α* for the scale was 0.87.

##### Self-Esteem

Self-esteem was measured using the Polish version (Łaguna et al., 2007) of the scale of Rosenberg (1965). The questionnaire consists of 10 items that pertain to individual self-worth and self-acceptance, with a 4-point response scale ranging from “1 = strongly disagree” to “4 = strongly agree.” Cronbach’s *α* for the scale was 0.82.

#### Measures in the Second Stage of the Study

##### Styles of Rumination

Intrusive and deliberate ruminations in the aftermath of the trauma were measured using the Event-Related Rumination Inventory (ERRI: Cann et al., 2011). The scale included 10 items assessing intrusive rumination and 10 items assessing deliberate rumination using a four-point scale from 0 to 3. Participants responded to two versions of each part of the scale. Firstly, they were asked about their ruminations during the weeks immediately after the trauma. Next, they responded to the same questions but about ruminations in the past 2 weeks. Cronbach’s *α* for intrusive rumination immediately after the event was 0.95, for intrusive rumination recently was 0.97, for deliberate rumination immediately after the event was 0.89, and for deliberate rumination recently was 0.95.

##### Coping Strategies

Coping with stress strategies was measured with the Brief Cope Scale (Carver, 1997). We asked the participants to identify their coping strategies for dealing with the traumatic experience they related in the interview. The questionnaire contains two items to measure each of the following 14 strategies: Self-Distraction, Active Coping, Denial, Substance Use, Use of Emotional Support, Use Of Instrumental Support, Behavioral Disengagement, Venting, Positive Reframing, Planning, Humor, Acceptance, Religion, and Self-Blame. Each of the items was rated on four-point response scale ranging from 1 (“I have not been doing this at all”) to 4 (“I have been doing this a lot”).

##### Symptoms of Anxiety and Depression

The presence and severity of anxiety and depression symptoms in the past week were assessed using the Hospital Anxiety Depression Scale (HADS: Zigmond and Snaith, 1983), a self-rating scale consisting of two subscales: HADS-A, comprising seven anxiety-related items and HADS-D, comprising seven depression-related items. Responses were given using a 0–3 scale. Cronbach’s *α* for the anxiety scale was 0.88, and for the depression scale was 0.81.

##### Life Satisfaction

Life satisfaction was measured using the Satisfaction with Life Scale (SWLS: Diener et al., 1985), adapted to Polish by Jankowski (2015). The measure asks the subject to agree or disagree, using a 7-point Likert-type scale (1 = strongly disagree, 7 = strongly agree), with five statements regarding the overall satisfaction with one’s life. Cronbach’s *α* for the scale was 0.90.

##### Posttraumatic Growth

In the study, we used the Polish translation of the Posttraumatic Growth Inventory (PTGI-X: Tedeschi et al., 2017). The scale consists of 25 items to be answered on a 6-point Likert scale, with values ranging from 0 (“I did not experience this change as a result of my crisis”) to 5 (“I experienced this change to a very great degree as a result of my crisis”), and assesses positive changes aftermath trauma on five areas: relating to others (seven items), new possibilities (five items), personal strength (four items), spiritual and existential change (six items), and appreciation of life (three items). Cronbach’s *α* for the PTGI-X was 0.95.

## Results

### Preliminary Analyses

In the first step of the analysis, we examined whether prioritizing positivity correlated with other study variables measured in the first stage. We also considered relationships between dependent variables: posttraumatic growth and life satisfaction.

As Table 1 shows, prioritizing positivity was moderately positively correlated with positive self-beliefs, i.e., hope and self-esteem. These results are consistent with previous cross-sectional studies (Catalino et al., 2014; Catalino and Boulton, 2020; Machlah and Zięba, 2021). Prioritizing positivity was also positively related to basic trust. Among the variables relating to a subject’s functioning in the period in which the second stage of the study was conducted, correlations between symptoms of depression and anxiety were shown, and moderately negative associations of these variables with life satisfaction. We found no significant associations between depression, anxiety and posttraumatic growth, which is consistent with the results of studies showing that PTG and symptoms of distress or disorder can, but do not always co-occur (Linley and Joseph, 2004; Shakespeare-Finch and Lurie-Beck, 2014). Posttraumatic growth, as in previous studies (Linley and Joseph, 2004; Durkin and Joseph, 2009) was positively associated with life satisfaction.

**Table 1:** Means, standard deviations, and correlations for pre-event and outcome variables.

Variable	*M*	*SD*	1	2	3	4	5	6	7
1. Prioritizing positivity	34.23	6.16							
2. Basic trust	29.98	5.45	0.27^**^						
3. Hope	48.33	8.16	0.23^**^	0.35^**^					
4. Self-esteem	28.83	4.65	0.20^*^	0.26^**^	0.58^**^				
5. Depression	5.12	3.86	0.02	0.07	−0.07	−0.26^**^			
6. Anxiety	8.59	4.60	−0.06	−0.00	−0.20^*^	−0.30^**^	0.70^**^		
7. Satisfaction with life	20.48	6.69	0.09	−0.00	0.31^**^	0.39^**^	−0.48^**^	−0.49^**^	
8. Posttraumatic growth	56.08	30.33	0.29^**^	0.19	0.16	0.01	−0.17	−0.15	0.28^**^

* *p* < 0.05;

** *p* < 0.01.

*M* and *SD* are used to represent mean and standard deviation, respectively.

### Prioritizing Positivity and Positive Beliefs as Predictors of Posttraumatic Growth and Satisfaction With Life

The results of the correlation analysis presented in Table 1 indicate relationships between the levels of variables measured before difficult events and the impact of these experiences on later participants’ functioning. Hope and self-esteem, i.e., positive beliefs about oneself, were negatively related to symptoms of depression and anxiety. These results seem to be consistent with many previous studies, which indicate that hope (Snyder, 1999, 2002; Gum and Snyder, 2002; Gallagher et al., 2020) and self-esteem (Watson et al., 1988; Łaguna et al., 2007) are conducive to experiencing positive emotions and reducing negative emotions. We obtained no results indicating a relationship between prioritizing positivity and the symptoms of depression and anxiety, and life satisfaction. However, in previous cross-sectional studies, prioritizing positivity showed positive correlations with satisfaction with life and negative relationships with depression and anxiety (Catalino et al., 2014; Machlah and Zięba, 2021). On the other hand, the level of prioritizing positivity measured before the traumatic or crisis experience seems to predict posttraumatic growth. In order to investigate the direct impact of prioritizing positivity and beliefs on the level of posttraumatic growth measured in the second stage of the study, we conducted linear regression analysis. The results are summarized in Table 2 and indicate that only prioritizing positivity was a significant predictor of posttraumatic growth among the analyzed variables.

**Table 2:** Regression results using posttraumatic growth as the criterion.

Predictor	*b*	*b* 95% CI (LL, UL)	*β*	*β* 95% CI (LL, UL)	*sr* ^2^	*sr*^2^ 95% CI (LL, UL)	*r*	Fit
Constant	−1.64	(−53.75, 50.48)						
Prioritizing positivity	1.20^*^	(0.19, 2.21)	0.25	(0.04, 0.47)	0.06	(−0.03, 0.15)	0.28^**^	
Basic trust	0.50	(−0.71, 1.71)	0.09	(−0.13, 0.32)	0.01	(−0.03, 0.04)	0.18	
Hope	0.50	(−0.48, 1.48)	0.14	(−0.13, 0.41)	0.01	(−0.03, 0.05)	0.14	
Self-esteem	−0.84	(−2.43, 0.75)	−0.14	(−0.39, 0.12)	0.01	(−0.03, 0.05)	0.01	
								*R*^2^ = 0.106^*^
								95% CI (0.00, 0.20)

* *p* < 0.05;

** *p* < 0.01.

A significant *b*-weight indicates the *β*-weight and semi-partial correlation are also significant. *b* represents unstandardized regression weights. *β* indicates the standardized regression weights. *sr*^2^ represents the semi-partial correlation squared. *r* represents the zero-order correlation. LL and UL indicate the lower and upper limits of a confidence interval, respectively.

Posttraumatic growth is a multidimensional construct and includes five areas of positive changes that may result from trauma (Tedeschi et al., 2018). Therefore, we performed additional linear regression analyzes in which the same variables as presented in Table 2 were predictors of specific posttraumatic growth’s areas. It turned out that prioritizing positivity was a statistically significant predictor of new possibilities: *β* = 0.29, *t* = 2.68, *p* = 0.009; and appreciation of life: *β* = 0.25, *t* = 2.34, *p* = 0.021. Additionally, hope was be the predictor of spiritual and existential changes: *β* = 0.29, *t* = 2.24, *p* = 0.028.

### Styles of Rumination and Coping Strategies as Predictors of Posttraumatic Growth and Satisfaction With Life

The results of the correlation analysis presented in Table 3 show that intrusive ruminations, both during the weeks immediately after the difficult event and recently, were negatively associated with life satisfaction, which is consistent with the results of previous studies (Triplett et al., 2012; Morgan et al., 2017). Deliberate ruminations, as in previous studies (Cann et al., 2011; Triplett et al., 2012; David et al., 2021; Taku et al., 2021), were positively associated with posttraumatic growth (overall result) and with posttraumatic growth experienced in the following areas: new possibilities, appreciation of life, and spiritual and existential changes.

**Table 3:** Means, standard deviations, and correlations between ruminations, coping strategies and outcome variables.

Variable	*M*	*SD*	SWL	PTG	PTG-PS	PTG-RO	PTG-NP	PTG-AL	PTG-SE
Intrusive ruminations 1	3.12	0.95	−0.20^*^	0.09	−0.66	0.18	0.14	0.05	0.34
Deliberate ruminations 1	3.47	0.80	−0.06	0.35^*^	0.21^*^	0.35^*^	0.36^*^	0.28^*^	0.28^*^
Intrusive ruminations 2	2.53	1.01	−0.29^**^	0.00	−0.19	0.06	0.05	0.00	0.04
Deliberate ruminations 2	2.89	0.99	−0.15	0.24^*^	−0.02	0.18	0.30^**^	0.30^**^	0.26^*^
Active coping	2.72	0.89	0.20^*^	0.37^**^	0.34^**^	0.32^**^	0.34^**^	0.29^**^	0.30^**^
Planning	2.70	0.84	0.11	0.30^**^	0.23^*^	0.25^*^	0.28^**^	0.23^*^	0.29^**^
Positive reframing	2.60	0.86	0.31^**^	0.24^*^	0.29^**^	0.18	0.12	0.16	0.28^**^
Acceptance	2.84	0.76	0.27^**^	0.12	0.15	0.07	0.01	0.09	0.18
Humor	2.09	0.97	0.23^*^	−0.12	−0.03	−0.10	−0.17	−0.09	−0.10
Religion	1.59	0.86	0.17	0.41^**^	0.33^**^	0.26^*^	0.35^**^	0.35^**^	0.51^**^
Use of emotional support	2.70	0.96	0.20^*^	0.16	0.14	0.26^*^	0.09	0.06	0.07
Use of instrumental support	2.60	0.97	0.18	0.13	0.16	0.20	0.06	0.02	0.06
Self-distraction	2.48	0.73	0.00	0.21^*^	0.10	0.23^*^	0.24^*^	0.19	0.13
Denial	1.54	0.71	−0.23^*^	0.06	−0.13	−0.02	0.22^*^	0.01	0.12
Venting	2.43	0.83	0.07	0.18	0.18	0.24^*^	0.18	0.11	0.06
Substance use	1.60	0.85	−0.07	0.05	−0.06	0.06	0.21^*^	−0.01	−0.01
Behavioral disengagement	1.74	0.75	−0.31^**^	0.08	−0.09	0.02	0.24^*^	−0.02	0.12
Self-blame	2.31	0.92	−0.26^**^	0.06	−0.05	0.04	0.19	0.01	0.04

* *p* < 0.05;

** *p* < 0.01.

*M* and *SD* are used to represent mean and standard deviation, respectively. SWL, satisfaction with life; PTG, posttraumatic growth; PTG-PS, personal strength; PTG-RO, relations to others; PTG-NP, new possibilities; PTG-AL, appreciation of life; and PTG-SE, spiritual and existential changes.

As Table 3 shows, some of the coping strategies were related to outcome variables. Active coping and positive reframing were positively correlated with life satisfaction and posttraumatic growth. Acceptance and seeking for emotional support seemed to predict life satisfaction, but not predict posttraumatic growth. On the other hand, religious coping was positively associated with posttraumatic growth but did not correlate with life satisfaction. Those results are partially in line with the results of meta-analysis by Prati and Pietrantoni (2009), according to which religious coping and positive reappraisal were strongly related to posttraumatic growth.

The results indicate that the role of specific coping strategies differs depending on which of the posttraumatic growth areas is concerned. The use of emotional social support was positively associated with the experience of posttraumatic growth only in the area of relationships with other people. Positive reframing seems to be related to an increasing sense of personal strength and spiritual and existential changes. Furthermore, the experience of posttraumatic growth in the area of new opportunities was fostered by using strategies that are usually considered maladaptive (Lazarus and Folkman, 1984; Carver et al., 1989): denial, substance use, and behavioral disengagement.

### Prioritizing Positivity and Positive Beliefs as Predictors of Styles of Rumination and Coping Strategies

The next step in the analysis was to check whether the prioritizing positivity, hope, basic trust, and self-esteem, measured before the difficult event, predicted engaging in deliberate and intrusive ruminations about a stressful experience and the use of particular coping strategies.

The results of the correlation analysis presented in Table 4 show that prioritizing positivity was positively associated with deliberate ruminations, both immediately after a difficult event and later. This seems to be consistent with the broaden-and-build theory of Fredrickson (1998, 2001) of positive emotions and with much previous evidence according to which induced positive emotions broaden the scope of thinking (Isen et al., 1987, 1991; Estrada et al., 1997; Fredrickson and Branigan, 2005). Prioritizing positivity was also related to active coping. This result can be explained because positive emotions have an undoing effect on negative emotional arousal and broaden an individual’s scope of action (Fredrickson, 2000, 2001).

**Table 4:** Means, standard deviations, and correlations between pre-event variables, ruinations and coping strategies.

Variable	Prioritizing positivity	Basic trust	Hope	Self-esteem
Intrusive ruminations 1	−0.05	0.11	−0.05	−0.09
Deliberate ruminations 1	0.20^*^	0.18	0.00	−0.16
Intrusive ruminations 2	0.14	0.25^**^	−0.05	−0.19
Deliberate ruminations 2	0.27^**^	0.25^*^	−0.02	−0.15
Active coping	0.24^*^	0.17	0.23^*^	0.17
Planning	0.08	0.09	0.16	0.07
Positive reframing	0.04	0.13	0.26^**^	0.21^*^
Acceptance	−0.17	0.00	0.11	0.13
Humor	0.02	−0.10	0.13	0.29^**^
Religion	0.09	0.16	0.21^*^	0.08
Use of emotional support	0.03	−0.03	−0.03	−0.02
Use of instrumental support	−0.04	−0.01	0.09	0.00
Self-distraction	0.02	0.12	−0.02	−0.05
Denial	0.04	−0.01	0.04	−0.12
Venting	0.03	0.08	−0.01	−0.06
Substance use	0.09	−0.12	−0.16	−0.14
Behavioral disengagement	0.08	0.12	−0.02	−0.19
Self-blame	−0.04	0.05	−0.09	−0.29^**^

* *p* < 0.05;

** *p* < 0.01.

Hope was positively related to coping with stress through positive reframing, which is consistent with the theory of hope of Snyder (2002). According to previous evidence, hope has a unique role in shaping positive appraisals of adversity and benefit finding from trauma or crisis (Snyder, 1999; Tennen and Affleck, 1999; Gum and Snyder, 2002). Hope was also positively related to religious coping. However, the results of study by Park (2006) did not confirm any connection between hope and religious coping, or between hope and stress-related growth.

### Styles of Rumination and Coping Strategies as Mediators of Beneficial Effect of Prioritizing Positivity and Positive Beliefs on Posttraumatic Growth and Life Satisfaction

We hypothesized that using adaptive coping strategies and deliberate ruminations would mediate the effect of prioritizing positivity, hope, basic trust, and self-efficacy on outcome variables. The results of the correlation and regression analysis presented earlier confirmed only some of our expectations regarding the relationship between predictors, coping strategies and styles of rumination, and the level of posttraumatic growth and life satisfaction. Therefore, in subsequent analyzes, we tested 12 mediation models for those variables for which we found significant correlations between predictors and outcome variables and between predictors and mediators. Using model 4 in the PROCESS macro, we examined the mediation hypotheses with bootstrap methods (Hayes, 2017). In each of the tested models, adding a particular style of rumination or coping strategy to the model as a potential mediator of the impact of the predictor on the level of posttraumatic growth or life satisfaction increased the size of the explanatory variance of the dependent variable but did not result in a statistically significant reduction in the direct impact of the predictor on the outcome variable. Therefore, none of the mediation hypotheses was confirmed.

In the next step of the analysis, we conducted three hierarchical linear regression analyzes for models that included: prioritizing positivity, hope, basic trust, self-efficacy, styles of ruminations, and coping strategies, as predictors for posttraumatic growth and life satisfaction. The results of these analyzes are presented in Tables 5, 6.

**Table 5:** Hierarchical regression results for posttraumatic growth.

Variable	*B*	*B* 95% CI (LL, UL)	*SE B*	*β*	*R* ^2^	Δ*R*^2^
Constant	9.37	(−24.71, 43.45)	17.14		0.08	0.08
Prioritizing positivity	1.33	(0.36, 2.29)	0.49	0.28^**^		
Constant	−17.50	(−56.16, 20.86)	19.12		0.15	0.07
Prioritizing positivity	1.07	(0.12, 2.02)	0.48	0.23^*^		
Deliberate ruminations—immediately	10.39	(2.90, 17.89)	3.77	0.28^**^		
Constant	−15.17	(−52.59, 22.24)	18.82		0.19	0.04
Prioritizing positivity	1.10	(0.17, 2.04)	0.47	0.23^*^		
Deliberate ruminations—immediately	14.38	(6.03, 22.73)	4.20	0.37^***^		
Intrusive rumination—recent time	−6.73	(−13.37, −0.08)	3.34	−0.22^*^		
Constant	−26.10	(−61.75, 9.55)	17.93		0.30	0.11
Prioritizing positivity	1.05	(0.17, 1.92)	0.44	0.22^*^		
Deliberate ruminations—immediately	12.48	(4.57, 20.39)	3.98	0.34^**^		
Intrusive rumination—recent time	−6.32	(−12,56, −0.07)	3.14	−0.21^*^		
Religious coping	11.33	(4.96, 17.70)	3.20	0.33^***^		

* *p* < 0.05;

** *p* < 0.01;

*** *p* < 0.001.

CI, confidence interval; LL, lower limit; and UL, upper limit.

**Table 6:** Hierarchical regression results for life satisfaction.

Variable	*B*	*B* 95% CI (LL, UL)	*SE B*	*β*	*R* ^2^	Δ*R*^2^
Constant	4.44	(−2.99, 11.87)	3.75		0.16	0.16
Self-esteem	0.55	(0.29, 0.80)	0.13	0.39^***^		
Constant	10.15	(1.78, 18.52)	4.22		0.21	0.06
Self-esteem	0.49	(0.24, 0.74)	0.13	0.35^***^		
Intrusive rumination—recent time	−1.60	(−2.76, −0.41)	0.60	−0.24^**^		
Constant	7.74	(−0.57, 16.06)	4.19		0.27	0.05
Self-esteem	0.42	(0.17, 0.66)	0.12	0.30^***^		
Intrusive rumination—recent time	−1.72	(−2.87, −0.56)	0.58	−0.26^**^		
Positive reframing	1.84	(0.49, 3.20)	0.68	0.24^**^		

** *p* < 0.01;

*** *p* < 0.001.

CI, confidence interval; LL, lower limit; and UL, upper limit.

According to the results of many previous studies, the predictors of posttraumatic growth in the study seems to be a relatively high intensity of deliberate rumination (in this case: immediately after the crisis event) and low intensity of intrusive rumination (in this case: during the last 2 weeks before the measurement, a few to several months after the crisis event). Researchers of posttraumatic growth argue that it is beneficial for the course of the posttraumatic re-adaptation process to gradually replace intrusive ruminations, the intensity of which is usually highest immediately after a traumatic event, with more reflective thinking characterizing deliberate ruminations (Cann et al., 2011; Tedeschi et al., 2018). The study results suggest that a higher level of posttraumatic growth was experienced by the respondents, who were more involved in reflective thinking in the initial period of coping with a crisis event and its consequences. Posttraumatic growth was also positively related to the relatively low intensity of recent intrusive thoughts.

The hierarchical linear regression analysis results in Table 5 suggest that a significant predictor of posttraumatic growth was prioritizing positivity measured before the crisis event. As already mentioned, the impact of prioritizing positivity was not mediated by any style of ruminations or coping strategy controlled in the study.

The results of the regression analysis presented in Table 6 indicate that life satisfaction had different predictors from posttraumatic growth. A significant predictor of life satisfaction was self-esteem. This is consistent with many previous studies relating positive associations between self-esteem, fulfillment of needs, achievement of life goals, and general satisfaction with life (Diener et al., 1985, 1999, 2003; Strobel et al., 2011). In the case of life satisfaction, the influence of recent intrusive thought was also significant, the higher intensity of which decreased the level of life satisfaction. The result may be explained by the fact that intrusive ruminations are associated with negative emotions, which may affect the assessment of life satisfaction (Cann et al., 2011).

## Discussion

The results of the prospective study presented and discussed above confirm that individual differences in prioritizing positivity can relate to a process of posttraumatic growth. Prioritizing positivity was associated with using an active coping strategy and deliberate but not intrusive ruminations. The mediation analysis results did not confirm the hypotheses that the impact of prioritizing positivity on posttraumatic growth is mediated by the intensification of deliberate ruminations and the use of adaptive coping strategies. Moreover, adding these variables to the model significantly improved the model’s fit but did not decrease the impact of prioritizing positivity on the variance of posttraumatic growth. Therefore, the question of how prioritizing positivity influences the course and outcome of the posttraumatic growth process remains open.

Previous studies on the role of prioritizing positivity have focused on the impact of seeking positive emotions in everyday life on the level of wellbeing (Catalino et al., 2014). Our results show that prioritizing positivity is an important personal resource in coping with stress and trauma. However, in the study, we did not control the extent to which differences in prioritizing positivity influenced the frequency and intensity of experiencing positive and negative emotions when the participants experienced a difficult or traumatic event and in the following weeks and months. Therefore, we do not know whether any differences occurred in the initial stage of posttraumatic adaptation, which is usually characterized by the highest intensity of stress and tension (Cann et al., 2011; Lelorain et al., 2012), and in later stages of dealing with the consequences of trauma. Arousing positive emotions in the first period could support the process of posttraumatic growth by reducing distress (Fredrickson et al., 2003) and thus lowering the intensity of automatic, intrusive thoughts. This would allow more reflective, deliberate cognitive processes that predict posttraumatic growth (Cann et al., 2011; Triplett et al., 2012). People with a high level of prioritizing positivity are characterized by the fact that they actively seek opportunities to experience positive emotions (Catalino et al., 2014; Catalino and Boulton, 2020). Prioritizing positivity, especially in the later stages of posttraumatic adaptation, could increase the readiness to see new opportunities to enjoy life and undertake new types of activities. That possibility is suggested by the obtained data, which show that prioritizing positivity was a predictor of positive changes mainly in these two areas of posttraumatic growth: new possibilities and appreciation of life. According to Van Cappellen et al. (2018), people who have a high level of prioritizing positivity may be better motivated to change their activity. Among the factors to consider when analyzing posttraumatic growth Jayawickreme et al. (2021) indicate the stability of behavior’s patterns. A traumatic experience can disrupt an individual’s habits and support discovering new ways of thinking and acting. However, those changes are rather temporary due to the tendency of most people toward the stability of habits. Therefore, prioritizing positivity may increase the readiness to change existing habits and behavior patterns by inducing positive emotions (Fredrickson, 2000, 2001; Fredrickson and Branigan, 2005). It could be conducive to developing and stabilizing positive posttraumatic changes in the long term. The aim of future research, which could be carried out using the diary research procedure, should therefore be to check the dynamics of changes in the area of experiencing positive emotions while coping with the consequences of trauma, as well as the possible impact of individual differences on prioritizing positivity.

Previous research shows that prioritizing positivity is positively associated with good relationships with other people (Catalino et al., 2014; Machlah and Zięba, 2021). Our results do not indicate that those study participants with a higher level of prioritizing positivity were more likely to seek social support as a coping strategy. Nonetheless, many studies show that social support is an effective and beneficial coping strategy (Carver et al., 1989; DeLongis and Holtzman, 2005; Aldwin, 2007). However, in the theoretical model of posttraumatic growth, the importance of a specific type of interpersonal relationship is emphasized, enabling positive disclosure rather than seeking social support to reduce stress symptoms (Calhoun and Tedeschi, 2013; Tedeschi et al., 2018). It will be worth conducting future research to check whether people with a high level of prioritizing positivity and thus having good interpersonal relationships use them to self-disclose and tell stories about their difficult experiences, favoring posttraumatic growth.

This study has limitations that must be taken into account. Firstly, the study group was quite diverse regarding the types of life event that participants reported as traumatic for them. In the study, we did not measure the level of posttraumatic stress symptoms or how the respondents rated the severity of the event. Only a small number of the participants in the first stage of the study (fever than ten people) refused to participate in the second stage, claiming that they had suffered no traumatic experience during this period. The other participants indicated their most difficult life events from the last 12 to 15 months. Perhaps not all of them would meet the definition of a seismic traumatic event used in the theory of posttraumatic growth (Tedeschi and Calhoun, 2004; Tedeschi et al., 2018). However, we followed the principle that what is traumatic varies from an individual point of view. Nevertheless, the study results should be interpreted bearing in mind that they relate to dealing with the consequences of difficult life events of varying severity.

Secondly, due to the study procedure, we could not control the influence of potential predictors of posttraumatic growth on decisions and choices made directly while dealing with trauma. The study’s strength is that the levels of prioritizing positivity, hope and basic trust were measured before the traumatic event. Thanks to this, we avoided some limitations typical of cross-sectional studies (Helgeson et al., 2006). However, potential mediators of the impact of these predictors on the level of posttraumatic growth, i.e., rumination and coping strategies, were only measured retrospectively in the second stage of the study, at the same time as the dependent variables.

It is also worth noting that the study participants reported as a traumatic event both events that were relatively distant in time, taking place about a year before the second stage of the study, and events that had taken place just a few months or weeks ago. Therefore, some of them may not have developed yet all the symptoms of posttraumatic growth (Bostock et al., 2009).

Furthermore, the study participants were diverse in age. Our results did not show statistically significant differences in the level of the analyzed variables due to age. Nevertheless, Manne et al. (2004) study showed an inverse relationship between age and posttraumatic growth. The ability to see the positive aspects of difficult experiences seems to increase with age. Moreover, the results of one study (Littman-Ovadia and Russo-Netzer, 2019) suggest age differences in the adaptive role of prioritizing positivity. Prioritizing positivity was more negatively associated with negative emotions for younger adults, but it was associated with more positive emotions among older individuals. It is also possible that different pathways in different age groups lead to posttraumatic growth or depreciation. Hence, an important direction in future researches may be exploring individual differences between younger adults and older adults in the process of posttraumatic growth.

Despite the limitations mentioned above, the study results provide new knowledge on the role of regulation of emotions in the process of posttraumatic growth and open up new research perspectives. These results can also be used to design effective psychological intervention techniques to support people experiencing trauma and psychological crises. As is known from recent research results, prioritizing positivity can be effectively developed through suitable microintervention (Van Cappellen et al., 2020). An essential purpose of clinicians supporting patients after experiencing trauma is to reduce the symptoms of posttraumatic stress. The current study results, like many other studies (Dickinson, 2021), also suggest the need to support positive changes after adversity. Clinicians should be open to the possibility of their patients perceiving positive consequences of trauma and support (not force) their occurrence. In psychoeducation, which is an element of therapy, knowledge should be shared about the role of positive emotions in dealing with the consequences of trauma and openness to new. Supporting the tendency to look for opportunities to experience positive emotions seems to be a remarkable opportunity to develop personal resources that increase people’s level of wellbeing and support them in the face of difficult and traumatic life events.

## Data Availability Statement

The raw data supporting the conclusions of this article will be made available by the authors, without undue reservation.

## Ethics Statement

The studies involving human participants were reviewed and approved by the Ethics Committee of SWPS University of Social Sciences and Humanities, Campus in Poznań. The participants provided their written informed consent to participate in this study.

## Author Contributions

MZ conceptualized the study and led the manuscript writing. NW and KW contributed to the study design, collected and analyzed the data, and wrote. MJZ contributed to the study design, analyzed the data, and wrote the manuscript. All authors contributed to the article and approved the submitted version.

## Funding

This study was supported by the grant 2013/10/E/HS6/00502 from the National Science Center, Poland, awarded to MZ.

## Conflict of Interest

The authors declare that the research was conducted in the absence of any commercial or financial relationships that could be construed as a potential conflict of interest.

## Publisher’s Note

All claims expressed in this article are solely those of the authors and do not necessarily represent those of their affiliated organizations, or those of the publisher, the editors and the reviewers. Any product that may be evaluated in this article, or claim that may be made by its manufacturer, is not guaranteed or endorsed by the publisher.
